# Mitochondrial lineage M1 traces an early human backflow to Africa

**DOI:** 10.1186/1471-2164-8-223

**Published:** 2007-07-09

**Authors:** Ana M González, José M Larruga, Khaled K Abu-Amero, Yufei Shi, José Pestano, Vicente M Cabrera

**Affiliations:** 1 Department of Genetics, Faculty of Biology, University of La Laguna, Tenerife 38271, Spain; 2 Department of Genetics, King Faisal Specialist Hospital & Research Center, Riyadh 11211, Saudi Arabia; 3 Department of Genetics, Faculty of Medicine, University of Las Palmas de Gran Canaria, Las Palmas 35080, Spain

## Abstract

**Background:**

The out of Africa hypothesis has gained generalized consensus. However, many specific questions remain unsettled. To know whether the two M and N macrohaplogroups that colonized Eurasia were already present in Africa before the exit is puzzling. It has been proposed that the east African clade M1 supports a single origin of haplogroup M in Africa. To test the validity of that hypothesis, the phylogeographic analysis of 13 complete mitochondrial DNA (mtDNA) sequences and 261 partial sequences belonging to haplogroup M1 was carried out.

**Results:**

The coalescence age of the African haplogroup M1 is younger than those for other M Asiatic clades. In contradiction to the hypothesis of an eastern Africa origin for modern human expansions out of Africa, the most ancestral M1 lineages have been found in Northwest Africa and in the Near East, instead of in East Africa. The M1 geographic distribution and the relative ages of its different subclades clearly correlate with those of haplogroup U6, for which an Eurasian ancestor has been demonstrated.

**Conclusion:**

This study provides evidence that M1, or its ancestor, had an Asiatic origin. The earliest M1 expansion into Africa occurred in northwestern instead of eastern areas; this early spread reached the Iberian Peninsula even affecting the Basques. The majority of the M1a lineages found outside and inside Africa had a more recent eastern Africa origin. Both western and eastern M1 lineages participated in the Neolithic colonization of the Sahara. The striking parallelism between subclade ages and geographic distribution of M1 and its North African U6 counterpart strongly reinforces this scenario. Finally, a relevant fraction of M1a lineages present today in the European Continent and nearby islands possibly had a Jewish instead of the commonly proposed Arab/Berber maternal ascendance.

## Background

The reconstruction of human history is a multidisciplinary objective. Alternative models proposed to explain the origin and dispersion of modern humans on the basis of paleoanthropological data [1] have received uneven support from other disciplines. From a genetic perspective, uniparental non-recombining markers have depicted the most complete and coherent picture of the origin of modern humans, clearly favoring the recent out-of-Africa hypothesis. The greatest diversity and the deepest phylogenetic branches for both Y-chromosome [2,3] and mtDNA [4,5] have been found in Africa. These African lineages have coalescence ages [6-9] compatible with a recent African origin of modern humans as proposed by fossil [10,11] and archaeological studies [12]. Furthermore, only more derived lineages have been found out of Africa supporting the hypothesis that, in their worldwide dispersion, modern humans replaced archaic humans inside and outside Africa. It seems that radiation in Africa of Y-chromosome M168 derived lineages [13] and L3 mtDNA lineages [14] preceded the out-of-Africa expansion. Focusing on mtDNA, all non-African lineages belong to two founder clusters, named M and N, which share a common root with their L3 African counterpart. Two possible out-of-Africa routes have been proposed: A southern coastal route bordering the Read Sea and an Eurasian continental route through the Levant. Based on mitochondrial phylogeography it was proposed that M lineages expanded with the coastal route to southern Asia and Oceania and N lineages by the continental route to Eurasia [7]. However, the posterior detection of primitive N lineages in southern areas as India [15,16] and Australia [6,17] weakened that hypothesis [18]. As, in addition, the founder ages of M and N are very similar, the alternative hypothesis, that M and N founders derived from a single African migration, was favored by several authors [16,19-21]. Another related disjunctive yet not settled is whether M and N (and its main branch R) arose inside or outside Africa [20]. The detection of a basal branch of haplogroup M in Africa (M1) gave support to the idea that haplogroup M originated in eastern Africa and was carried towards Asia with the out-of-Africa expansion [22]. The alternative hypothesis, that haplogroup M1 could trace a posterior backflow to Africa from Asia, considered by several authors [7,21,23,24] has not yet gained experimental support because, until now, no ancestral M1 lineages have been found outside Africa [21,24,25].

To shed light on this haplogroup we have constructed a phylogeny of the M1 clade based on the analysis of 13 complete or nearly complete mitochondrial sequences representing the main branches of M1 and realized a phylogeographic study using 261 partial M1 sequences to determine the most probable age and origin of this clade and the temporal and spatial frame of its secondary expansions in Africa and Eurasia.

## Results

Although we have only completely sequenced a limited number of M1 lineages, the combination of HVSI haplotypes and RFLP status carried out for the rest of our M1 samples allow us to be confident that we have not missed any new basic M1 lineage.

**Figure 1:**
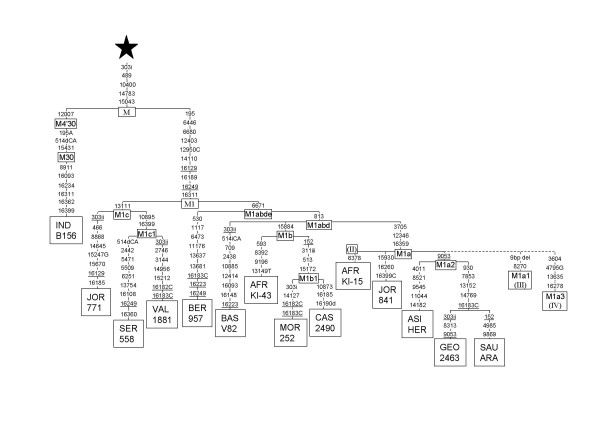
**Phylogenetic tree based on complete M1 sequences**. Numbers along links refer to nucleotide positions. C, G indicate transversions; "d" deletions and "i" insertions. Recurrent mutations are underlined. Star differs from rCRS [62, 63] at positions: 73, 263, 311i, 750, 1438, 2706, 4769, 7028, 8701, 8860, 9540, 10398, 10873, 11719, 12705, 14766, 15301, 15326, 16223 and 16519. Subject origins are: Asian (ASI HER; [54]) and 2 Ethiopians (AFR-KI43 and AFR-KI15; [55]) only analyzed for coding region; Georgian (GEO 2463); Indian (IND-B156; [25]); 2 Jordanians (JOR 771; [7] and JOR 841); 2 Moroccans (MOR 252; [7] and BER 957 = Berber); Saudi Arab (SAU ARA); Serere from Senegal (SER 558); 3 Spanish (Basque = BAS V82, Castilian = CAS 2490, and Valencian = VAL 1881). Doted branches include subjects only analyzed for RFLP and HVI region [22]. Roman numbers refers to the Quintana-Murci et al. [22] nomenclature.

As an outgroup of the M1 genomic phylogenetic tree (Fig. 1) we used a published Indian M30 complete sequence [25]. When this M30 lineage is compared to the rare M sequence previously detected in two Palestinians [26], it is evident that it belongs to the Indian super-clade M4'30, as it shares the basal mutation 12007. More specifically it belongs to the M30 branch because it also has transition 15431. M30 has a broad geographic, ethnic and linguistic range in India. It has been detected in northern and southern India, in Australoid and Caucasoids, and in Dravid and Indo-European speakers [24,25]. So, instead of an autochthonous Near East M lineage, its presence in Palestine is probably due to a recent gene flow from India. After careful re-reading and partial re-sequencing of two previously published M1 sequences [7], we have detected in them the following errors: both have the 12950C transversion, and, in addition M1,1 has the 6671 transition and M1,2 the 13111 transition. Taking these modifications into account, from the M basal type, haplogroup M1 is characterized by one transversion (12950C) and four transitions (6446, 6680, 12403, and 14110) in the coding region and by a five transitions motif (195, 16129, 16189, 16249, and 16311) in the non-coding region (Fig. 1). This haplogroup can be RFLP diagnosed by a *Mnl*I site loss at position 12402. Two main branches, M1c and M1abde, respectively defined by transitions 13111 and 6671, sprout from the root. Based on partial sequences M1c was defined by transition 16185 [21]. However, not all M1c lineages present this mutation that, in addition, recurrently appears in a M1b1 lineage. It seems that for population studies M1c could be better diagnosed by a *Dde*I site loss using a modified reverse primer (Table 1). It is surprising that none of the three M1c complete sequences have an eastern Africa ancestry: one (Jor771) has a Levantine origin and the other two belong to West sub-Saharan Africa (SER558) and West Mediterranean (VAL1881) areas. The latter two sequences conform a new M1c1 subclade defined by transitions 10895 and 16399 that can be RFLP diagnosed at 10895 position (Table 1). In relation to the M1abde cluster, it is also surprising that one lineage that directly branched out from the root (BER957) has a northwestern, not eastern, Berber ancestry. All the rest of lineages shared the 813 transition forming the M1abd cluster. Again, an isolate offshoot of Basque ancestry (BASV82) sprouts from its root. Subclade M1b was characterized by an RFLP site gain (+15882 *Ava*II) and loss of -15883 *Hae*III [22]. Later an M1b subclade defined by the non-coding motif 16260–16320 and restricted to East Africans was identified [21]. Consistently, none of our M1b sequences from western areas has that motif. The last cluster, M1a, was first distinguished by RFLP +12345 *Rsa*I [22] and, after that, further characterized by transition 16359 [21]. In addition it also has transition 3705 at its root (Fig. 1). M1a is the most prominent clade in eastern Africa. However, its expansion occurred later than the other M1 branches (Fig. 1). An M1a subclade, M1a2, defined by transition 9053, that can be RFLP diagnosed (Table 1), testifies a posterior spread of M1a to western Asia.

**Table 1:** Diagnostic RFLPs for different M1 clusters

Cluster	Diagnostic RFLP
M1	-12402 Mnl I ^(1) ^+12950 Aci I, +14110 Ear I
M1a	+12345 RsaI ^(2)^
M1b	-15883 HaeIII/+15882 Ava II ^(2)^
M1c	-13110 Dde I ^(3)^
M1a2	-9052 Hae II/-9053 Hha I
M1b1	-15172 Hae III
M1c1	+10893 Taq I

^1 ^= [18]

^2 ^= [21, 22]

^3 ^= The primers used in the amplification were: L13069 = 5' AggCCCCACCCCAgTCTCAg 3' and the modify H13112* = 5' gCTAgggggTggAAgCggATgACTA 3'

### Geographic distribution of M1

**Figure 2:**
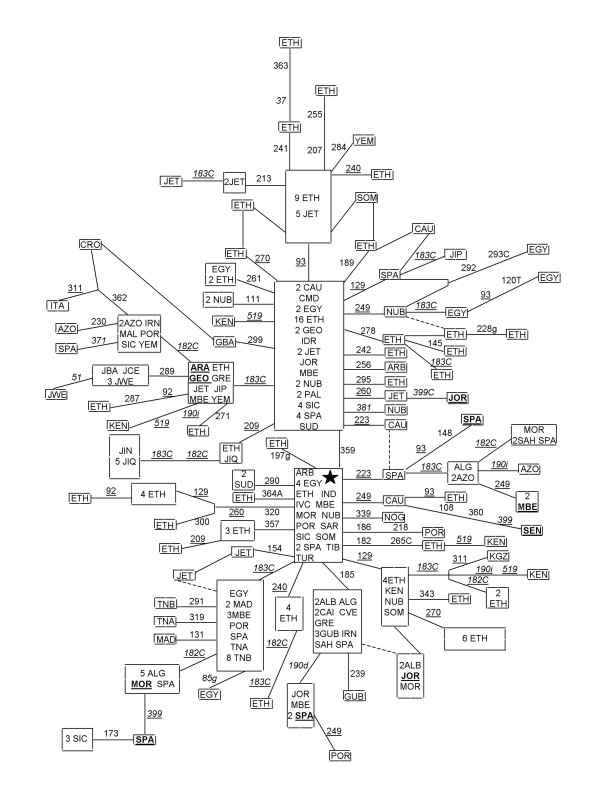
**Reduced median network relating M1 HVSI sequences**. The central motif (star) differs from rCRS at positions: 16129 16189 16223 16249 16311 for HVI control region. Numbers along links refer to nucleotide positions minus 16000: homoplasic mutations are underlined, and positions not used in diversity estimations are in italics. The broken lines are less probable links in accordance with completed sequences (Fig. 1) and/or mutation recurrence. Size of boxes is proportional to the number of individuals included. Codes are: NWA = Northwest Africa (ALB = Algerian Berber; ALG = Algerian; MBE = Moroccan Berber; MOR = Moroccan; SAH = Saharan; TNA = Tunisia Arab; TNB = Tunisia Berber); CWA = Central West Africa (GUB = Guinea Bissau; IVC = Ivory Coast; MAL = Mali; SEN = Senegalese); NEA = Northeast Africa (EGY = Egyptian; NUB = Nubian; SUD = Sudanese); CEA = Central East Africa (ETH = Ethiopian; KEN = Kenyan; SOM = Somali); WAS = West Asia (ARA = Arab; ARB = Arab Bedouin; CAU = Caucasian; GEO = Georgian; JOR = Jordanian; IDR = Israel Druze; IND = Indian; IRN = Iranian; KGZ = Kirghiz; NOG = Nogay; PAL = Palestinian; TIB = Tibetan; TUR = Turkish; YEM = Yemeni); IPE = Iberian Peninsula and islands (AZO = Azores; CAI = Canary Islander; CVE = Cape Verde; MAD = Madeira islander; POR = Portuguese; SPA = Spanish); MEU = Mediterranean Europe (CRO = Croatian; CMD = Central Mediterranean; GRE = Greek; ITA = Italian; SAR = Sardinian; SIC = Sicilian); REU = Rest of Europe (GBA = English); JEW = Jews (JBA = Baltic Jew; JCE = Central Europe Jew; JET = Ethiopian Jew; JIQ = Iraqi Jew; JIN = Iranian Jew; JIP = Spanish Jew; JWE = Western Europe Jew). In boldface and underlined individual complete sequenced.

Figure 2 shows the reduced median network obtained from the 261 M1 haplotypes found in a global search comprising more than 38,713 HVSI sequences. In Africa, haplogroup M1 has supra-equatorial distribution (see additional files 1 and 2). As previously reported its highest frequencies and diversities (Table 2) are found in Ethiopia in particular and in East Africa in general. Two appreciable gradients exist. Frequencies significantly diminished from East to West and also going South to sub-Saharan areas. M1 is not uncommon in the Mediterranean basin showing a peak in the Iberian Peninsula. However, it is rare in continental Europe. Although in low frequencies, its presence in the Middle East has been well established from the South of the Arabian Peninsula to Anatolia and from the Levant to Iran. The central HVSI haplotype (16129–16189–16223–16249–16311) has been found only once in northwestern India [27]. Another possible Indian M1 candidate is the derived sequence: 16086–16129–16223–16249–16259–16311 [28]. However, in two recent studies in which 24 [24] and 56 [25] Indian M complete sequences were analyzed no ancestral M1 lineages have been found. M1 haplotypes have also been occasionally spotted in the Caucasus and the Trans Caucasus [23,29] and in Central Asia [30]. It seems that, going east, M1 even reached the Tibet as the HVSI diagnostic motif was sampled there [31]. However, although haplotypes sharing four of the five HVSI transitions defining M1 (16129–16223–16249–16278–16311–16362; 16129–16223–16234–16249–16311–16362) have been sampled in Thailand and Han Chinese [32,33], complete sequencing have unequivocally allocated them in the D4a branch of D, the most abundant haplogroup representing M in East Asia. As commented previously, this is a clear example of the danger of establishing affinities between geographically distant areas only on the basis of HVSI homologies as, often, they are the product of geographic isolation and molecular convergence [18]. Within this sparse but geographically wide range of M1 distribution its three identified branches also had uneven radiations. Although M1a (HVSI identified by the 16359 transition) is present in all the M1 range, its greatest frequencies and diversities are found in Ethiopia and eastern Africa (Table 2), pointing to this area as the most probable origin of the M1a expansion in all directions, with particular incidence in western Asia and sub-Saharan Africa. Not all the M1b lineages can be HVSI identified; however, several specific subclades have different locations. Those characterized by transitions 16260–16320 [21], and by presence of 16182 transition and 16265C transversion [22] are restricted to Ethiopia with occasional spreads to eastern Africa. In addition, there is an M1b branch, identified by 16185 transition and 16190 deletion that has a northwestern distribution excepting a Jordan haplotype (Fig. 2). Despite that M1c cannot be unequivocally defined by transition 16185, it can be stated that M1c is an overwhelmingly Northwest African clade which spreads to the Mediterranean and West sub-Saharan Africa areas. Finally, other unclassified M1 branches have also different geographic ranges. Those identified by the presence of 16357 transition and by the reversion of the diagnostic position 16129 are of Ethiopian eastern Africa adscription, while clusters characterized by loss of the diagnostic position 16223 and by the 16399 transition have a northwestern distribution (Fig. 2). However, M1 assignation of haplotypes, which lack any of the basic positions, based only on HVSI information is risky when they share other diagnostic positions with different haplogroups. For instance, the Russian haplotype 16183C–16189–16249–16311, classified as M1 on the basis of its HVSI sequence [34] also matches with haplotypes assigned to the U1a clade [35].

**Table 2:** Total of individuals sampled and frequencies, nucleotide diversity and gene diversity for M1 and M1a clusters. a) local, b)Jew and c) total individuals.

a)	**Locals**								
name^a^	sample	nM1^b^	%M1	pi*1000	h(%)	nM1a	%M1a	pi*1000	h(%)
*Europe*									
IPE	5007	34	0.7	5.6 ± 3.8	84 ± 3	10	29	1.4 ± 1.7	38 ± 18
MEU	3278	15	0.5	5.5 ± 3.9	76 ± 10	9	60	3.0 ± 2.7	42 ± 19
REU	10735	1	0.0	-	-	1	100	-	-
TEU	19020	50	0.3	5.8 ± 3.9	84 ± 3	20	40	2.5 ± 2.2	45 ± 14
*Africa*									
NWA	1175	38	3.2	3.9 ± 2.9	70 ± 7	2	5	0.0 ± 0.0	0 ± 0
CWA	2351	7	0.3	7.2 ± 5.3	86 ± 14	1	14	-	-
NEA	288	23	8.0	5.7 ± 3.9	85 ± 5	13	57	4.9 ± 3.7	78 ± 10
ETH	344	53(78)	15.4	9.4 ± 5.6	92 ± 2	45	58	5.5 ± 3.7	82 ± 5
CEA	533	61(86)	11.4	9.1 ± 5.5	92 ± 2	48	56	5.4 ± 3.7	81 ± 5
WA	3526	45	1.3	4.5 ± 3.2	75 ± 6	3	7	0.0 ± 0.0	0 ± 0
EA	821	84(109)	10.2	8.5 ± 5.2	92 ± 2	61	56	5.5 ± 3.7	81 ± 5
SEA	4347	129(154)	3.0	7.9 ± 4.9	91 ± 1	64	42	5.2 ± 3.6	79 ± 5
*Asia*									
WAS	7589	36(28)	0.5	5.5 ± 3.8	78 ± 8	18	64	2.8 ± 2.4	49 ± 14
*Total*									
TOT	30956	215(232)	0.7	7.2 ± 4.5	89 ± 1	102	44	4.4 ± 3.1	69 ± 5
EAs	5912	0	0.0	-	-	-	-	-	-
SAf	596	0	0.0	-	-	-	-	-	-
									
b)	**Jews**								
name^a^	sample	nM1^b^	%M1	pi*1000	h(%)	nM1a	%M1a	pi*1000	h(%)

*Europe*									
JIP	48	2	4.2	3.6 ± 5.1	100 ± 50	2	100	3.6 ± 5.1	100 ± 50
JRE	663	6	0.9	0.0 ± 0.0	0 ± 0	6	100	0.0 ± 0.0	0 ± 0
JTE	711	8	1.1	2.5 ± 2.4	46 ± 20	8	100	2.5 ± 2.4	46 ± 20
*Africa*									
JNWA	135	0	0.0	-	-	-	-	-	-
JNEA	20	0	0.0	-	-	-	-	-	-
JET	69	14	20.3	5.7 ± 4.0	81 ± 7	12	86	3.8 ± 3.1	76 ± 8
JCEA	69	14	20.3			12	86		
JEA	89	14	15.7			12	86		
JSEA	224	14	6.3			12	86		
*Asia*									
JAS	473	7	1.5	0.0 ± 0.0	0 ± 0	7	100	0.0 ± 0.0	0 ± 0
*Total*									
JEW	1408	29	2.1	6.3 ± 4.2	86 ± 3	27	93	5.6 ± 3.8	84 ± 3
									
c)	**Totals**								
name^a^	sample	nM1^b^	%M1	pi*1000	h(%)	nM1a	%M1a	pi*1000	h(%)

*Europe*									
IPEj	5055	36	0.7	5.7 ± 3.8	84 ± 3	12	33	1.7 ± 1.8	44 ± 16
REUj	11398	7	0.1	2.1 ± 2.2	29 ± 20	7	100	2.1 ± 2.2	29 ± 20
TEUj	19731	58	0.3	6.2 ± 4.1	86 ± 3	28	48	3.3 ± 2.6	64 ± 9
*Africa*									
NWA	1310	38	2.9			2	5		
NEA	308	23	7.5			13	57		
ETHj	413	67(92)	16.2	9.1 ± 5.5	92 ± 2	57	62	5.4 ± 3.6	82 ± 4
CEAj	602	75 (100)	12.5	8.9 ± 5.4	92 ± 2	60	60	5.3 ± 3.6	81 ± 4
EAj	910	98(123)	10.8	8.4 ± 5.1	92 ± 2	73	59	5.4 ± 3.7	82 ± 4
SEAj	4571	143(168)	3.1	8.0 ± 4.9	92 ± 1	78	45	5.5 ± 3.7	81 ± 4
*Asia*									
WASj	8062	43(35)	0.5	5.7 ± 3.9	82 ± 5	25	71	3.6 ± 2.3	67 ± 9
*Total*									
TOTj	32364	244(261)	0.8	7.4 ± 4.6	90 ± 1	129	49	4.8 ± 3.3	77 ± 4

^a ^Name for locals, Jews and total for different areas: Europe: Iberian Peninsula and related Islands (IPE, JIP, IPEj), Europe Mediterranean area (MEU), Rest of Europe (REU, JRE, REUj), and total Europe (TEU, JTE, TEUj); Africa: Northwest Africa (NWA, JNWA), Central West Africa (CWA), Northeast Africa (NEA, JNEA), Ethiopia (ETH, JET, ETHj), Central East Africa (CEA, JCEA, CEAj), East Africa (EA, JEA, EAj), West Africa (WA) and Supra-Equatorial Africa (SEA, JSEA, SEAj); Asia: West Asia (WAS, JAS, WASj); Total of these samples (without South Africa and East Asia) (TOT, JEW, TOTj); East Asia (EAs); South Africa (SAf).

^b ^Number outside and inside the parenthesis were used to estimate the M1 and M1a frequency, respectively.

The presence in the Mediterranean basin and in West sub-Saharan Africa of M1a and M1c lineages can be taken as proof that these areas received influences both from the West and East North African centers of M1 radiation. Quantitative confirmation of the above described patterns are provided by AMOVA and pairwise distances based on F_ST _analyses using the groups and populations described in Material and Methods and taking into account haplotypic molecular differences. As usual the bulk of the variation, 90%, is within populations, 6% is due to differences among groups and 4% to differences among populations within groups. Pairwise differences between populations (Table 3) offer a more detailed view. There is homogeneity between populations within eastern Africa, small differences (p < 0.05) within western Africa and strong heterogeneity between these main areas (p < 0.001). On the contrary, Iberian Peninsula has significant differences with the rest of Europe. In turn, West Asia conforms an homogenous continuum with East Africa and Europe excepting Iberian Peninsula and the latter is not significantly different of western Africa. All these results can be explained as due to the differential radiation of M1a from East Africa and M1c from Northwest Africa, the Iberian Peninsula being mostly influenced by Northwest Africa and the rest of Europe and western Asia by East Africa.

**Table 3:** Population pairwise F_ST_s based on M1 haplotypes.

Populations	MEU	IPE	NWA	CWA	NEA	CEA	WA	EA	WAS
MEU	-	0.09*	0.31***	0.27**	0.03	0.05*	0.28***	0.04*	0.01
IPE		-	0.02	0.08	0.09**	0.09***	0.02	0.08***	0.08**
NWA			-	0.12*	0.26***	0.18***	0.00	0.18***	0.27***
CWA				-	0.26**	0.20**	0.05	0.21***	0.26**
NEA					-	0.02	0.24***	0.01	0.00
CEA						-	0.19***	0.00	0.02
WA							-	0.18***	0.25***
EA								-	0.01
WAS									-

* = p < 0.05; ** = p < 0.01; *** = p < 0.001

Abbreviated name populations as in table 2

### M1 haplotypes in Jews

Several M1 haplotypes have been detected in Jewish communities albeit in low frequencies [36,37]. However, when compared with non-Jew populations they show significantly higher frequencies for the whole M1 haplogroup (p = 33.54***) and for M1a in particular (p = 24.90***). The only striking exception is that of the Moroccan Jews for which no M1 lineages have been detected at all [36]. Interestingly, all M1 lineages found in Jews, except two, belong to the eastern clade M1a (Fig. 2). Therefore, as for the bulk of the M1 Near East haplotypes, the most probable origin of these Jewish M1 lineages is the result of an eastern African expansion around 5000 years ago. Another peculiarity of M1 in Jewish communities is its reduced haplotypic diversity (Table 2) which has been already detected for other mtDNA lineages [36,38]. In addition, there is a strong M1 geographic differentiation among Jewish communities. For example, all European Ashkenazi Jews have only one M1a lineage characterized by a transition in the 16289 position that has not been detected in other Jew or non-Jew populations. Similarly, all West Asian Jews shared an identical M1a motif characterized by a transition in the 16209 position that has been detected only once in Ethiopia. These results are congruent with the proposition that, in the majority of the cases, Jewish migrations implied strong maternal founder effects [36-38]. Nevertheless, as M1a Jewish lineages are unique and different in different groups, we think that its source Near East population should not suffer strong genetic bottlenecks. Finally, it is worth mentioning that M1 frequencies of Jewish groups and their host populations are significantly correlated (r = 0.942**) which suggests that some genetic interchange must have happened between them as already proposed by others authors [36,37].

### Radiation ages and evolution of lineages

Radiation ages for M1 and its subhaplogroups have been estimated on the basis of complete coding and HVSI sequences using different mutation rate estimations (Table 4). The ages obtained for M1 and M1a from HVSI data are more coherent with those calculated for the coding region using the Ingman et al. [6] mutation rate than that proposed by Mishmar et al. [8]. Our coalescence age estimations for the whole M1 clade (20,000–30,000 years) are younger than those previously published [22]; however, the approximate expansion ages for the eastern Africa M1a subclade (10,000–20,000 years) are in the same range. Although standard errors overlap, it seems that the northwestern Africa expansion represented by M1c subclade (19,040 ± 4916 years), preceded the M1a eastern Africa expansion (16,756 ± 5997) M1b being the youngest branch (10,155 ± 3590). It must be stated that coalescence ages are only rough estimations biased by mutation rate estimations, small sample size, demographic history and, possibly, selection. There are recent examples of clock-like evolution violations in several mtDNA lineages that have been explained by selective or demographic effects [39-41]. Here, subclade M1a2 (Fig. 1) represents a new example of constant mutation rate violation. The mean number of substitutions accumulated in M1a2 lineages (12.5 ± 0.7) is significantly higher (p = 0.008) than that in the rest of M1 lineages (8.4 ± 1.3). This result is not compatible with a uniform rate of evolution. The small standard errors show that there is high lineage homogeneity within groups, which weakens the possibility that stochastic processes have played a main role. Different patterns of synonymous and nonsynonymous changes among different lineages have been taken as hints of a role for selection in other studies [8,39]. In our case differences between synonymous vs. nonsynonymous changes within groups does not reach statistical signification (p = 0.75). However, the mean number of coding region substitutions accumulated in M1a2 lineages (11 ± 0.0) is significantly higher (p < 0.001) than in the rest of M1 (5.6 ± 0.7). Conversely, the mean number of regulatory region substitutions accumulated in M1a2 lineages (1.5 ± 0.7) is smaller than in the rest (2.8 ± 0.9) although not reaching statistical significance (p = 0.175). If the mutation rate was constant along the whole mtDNA molecule, for each mutation in the regulatory region roughly fourteen mutations should accumulate in the coding region. However, selection pressure is higher in the coding than in the regulatory region so that the substitution rate is ten times faster in the latter. The mean coding/regulatory ratio is 8.3 for M1a2 lineages and only 2.4 for the rest of M1. We interpret these results as due to different ages of expansion between clades. M1a2 would be the youngest clade with a more recent expansion than the others so that purifying selection has not had enough time to eliminate mutations with small deleterious effects in the coding region. We think that differences in the rate of evolution among subgroups of the North African U6 haplogroup [40] could be better explained by the same pattern assuming that the U6a subclade, with the highest coding/regulatory ratio, had a more recent radiation than the U6b subclade. In spite of its anomalous behavior, M1a2 has only a minor effect on the estimation of the whole M1 coalescence age although its omission significantly diminishes that of the M1a subgroup (Table 4).

**Table 4:** Estimated ages (years) for different subgroups of M1 haplogroup, based on coding and HVSI regions.

	Complete sequences					HVI sequences	
		
	Coding region			HVSI			
Subgroup	N	age^a^	age^b^	N	age	N	age
M1	13	26071 ± 5297	35175 ± 7147	10	30270 ± 11767	261	26365 ± 13319
M1witout a2	10	21706 ± 4457	29286 ± 6014	8	32793 ± 12358		
M1c	3	19040 ± 4916	25689 ± 6633	3	33633 ± 15041		
M1b	3	10155 ± 3590	13701 ± 4844	2	-		
M1a	5	16756 ± 5997	22607 ± 8091	3	6727 ± 6727	129	14236 ± 5124
M1a without a2	2	3808 ± 2693	5138 ± 3633	1	-		

a.- mutation rate of Ingman et al. [6]

b.- mutation rate of Mishmar et al. [8]

## Discussion

### Phylogeographic parallelism between M1 and U6 haplogroups

There are striking similarities between the geographical dispersals and radiation ages observed here for M1 lineages and those previously published for the North African U6 haplogroup [40]. It was proposed that U6a first spread was in Northwest Africa around 30,000 ya. Coalescence ages for M1 also fit into this period and the oldest clade M1c has an evident northwestern Africa distribution; however it had to have a wide geographic range as some M1c lineages are today still present in Jordanians (Figs. 1 and 2). It is curious that this prehistoric Near Eastern colonization was also pointed out by the uniqueness of the U6a haplotypes detected in that area. A posterior East to West African expansion around 17,000 ya was indicated by the U6a1 relative diversity and distribution. Again, age, relative East to West diversities and geographic range accurately correspond with the M1a1 expansion detected here. More recent local spread of lineages U6b and U6c also parallel the M1b and M1c1 distributions. Furthermore, these similarities also hold outside Africa. U6 lineages in the Iberian Peninsula have been considered traces of northward expansions from Africa. Based on the uneven distribution of U6a and U6b lineages in Iberia, with the former predominating in southern and the latter in northern areas, it was proposed that U6b in Iberia represents a signal of a prehistoric North African immigration whereas the presence of U6a could be better attributed to the long lasting historic Arab/Berber occupation [40]. Again, this pattern is accurately repeated by the M1c and M1a distribution in the Iberian Peninsula, the northwest African M1 being more abundant in northern areas (56%) and the East African M1a in southern areas (85%) although, due to the small sample size, difference does not reach a significant level (p = 0.07). Additional support to the hypothesis of a prehistoric introduction are the recently detected presence of a Northwest African M1c lineage in a Basque cemetery dated to the 6^th^–7^th ^centuries AD, prior to the Moorish occupation [42], and the ancestral phylogenetic position of another Basque M1d sequence (Fig. 1) that does not match any African sequence. Finally, two autochthonous U6 lineages (U6b1 and U6c1) traced the origin of the Canary Islands prehispanic aborigines to Northwest Africa [43]. Although exclusive M1 lineages have not been detected in the Canary Islands, it is worth mentioning that those sampled belong to the Northwest African area [44]. Outside Africa and the Iberian Peninsula, as with U6, M1 has been mainly detected in other Mediterranean areas with main incidences in islands such as Sicily. It is customary to attribute these incidences to the above mentioned Arab/Berber historic occupations. However, taking into account the major Jewish assignation for all the M1a haplotypes detected in Europe, the possibility of a Jewish maternal ascendance for at least some of these lineages should not be rejected.

Note that the two M1 lineages sampled in the Balearic isles were of Jewish adscription [45]. Also, there were well documented Jewish settlements in Sicily since early Roman times [46] and, coincidentally, half of the M1 lineages sampled in that island [47,48] belong to the M1a cluster. Finally, the Atlantic archipelagos of Canaries and Madeira, where the rigor of the Spanish Inquisition was stronger, only have M1c representatives. In contrast, in the Azores Islands, that were used as a refuge by Sephardim Jews expelled from the Iberian Peninsula, half of the M1 sequences detected are of M1a assignation [49,50]. These possible Jewish contributions might be also extended to the U6 lineages of eastern origin because all U6 haplotypes detected in Ashkenazim and other Jewish groups, excepting one that is a basal U6a (16172–16219–16278), belong to the eastern Africa clade U6a1 [36,26]. An additional proof of the striking parallelism between M1 and U6 lineages is the fact that, as for M1, no U6 representatives were sampled in Moroccan Jews in spite of the high frequency of this clade in the Moroccan and Berber host populations [36].

### Most probable origin of M1 ancestors

Mitochondrial M lineages in Ethiopia were first detected by RFLP analyses [51]. To explain its presence in that area the authors suggested two possibilities: 1) the marker was acquired by Ethiopians through interchanges with Asians or 2) it was present in the ancient Ethiopian population and was carried to Asia by groups who migrated out of Africa. Later, the second hypothesis was favored and a single origin of haplogroup M in Africa was suggested, dating the split between Asian and African M branches older than 50,000 ya [22]. Although not completely discarding this last scenario other authors considered that the disjunctive was unsettled. The vast diversity of haplogroup M in Asia compared to Africa pointed to the possibility that M1 is a branch that traces a backflow from Asia to Africa [7,23]. Due to the scarcity of M lineages in the Near East and its richness in India, this region was proposed as the most probable origin of the M1 ancestor [7,52]. However, recent studies based on Indian mtDNA sequences [24,25] have not found any positive evidence that M1 originated in India. Nevertheless, the inclusion of M1 complete mtDNA lineages in the construction of the macrohaplogroup M phylogeny clearly established that the antiquity of Indian lineages, as M2, as compared to Ethiopian M1 lineages support an Asian origin of macrohaplogroup M [24]. Furthermore, the comparison within Africa of eastern and western M1 sequences left the origin of M1 in Africa uncertain [21]. On the light of our and other authors results, it seems clear that by their respective coalescence ages and diversities, M1 is younger than other Asiatic M lineages. Although it is out of doubt that the L3 ancestor of M had an African origin, macrohaplogroup M radiated outside Africa and M1 should be considered an evolved branch that signals its return to this continent. Even more, as the coalescence ages of the northwestern M1c clade is older than the eastern M1a clade, we think that the most ancient dispersals of M1 occurred in northwestern Africa, reaching also the Iberian Peninsula, instead of Ethiopia. The detection of an ancestral M1c sequence in Jordanians could be explained by two alternative hypotheses: 1) that the Near East was the most probable origin of the primitive M1 dispersals, West into Africa and East to Central Asia. This supposition would explain the presence of basic M1 lineages, instead of the most common M1a derivates, as far as the Tibet. The actual scarcity of these types in eastern areas could be explained by posterior migrations that erased these primitive lineages. The absence of these ancestral M1c lineages in Ethiopia would point to the Sinai Peninsula as the most probable gate of entrance of this backflow to Africa. 2) That M1 is an autochthonous North African clade that had its earliest spread in northwestern areas marginally reaching the Near East and beyond. This would explain the shortage of basic M1 lineages in the Near East but would leave the Asiatic origin of the M1 ancestor undetermined. In any case, both alternatives envisaged M in Africa as an offshoot of the Asiatic M trunk. The striking phylogeographic parallelism between U6 and M1 haplogroups adds additional support to these hypotheses. It is possible to correlate the dispersion ages of the different M1 clades with their contemporary climatic, archaeological, paleoanthropological and linguistic information. For instance, the first M1 backflow to Africa, dated around 30,000 ya, is coincidental with a harsh glacial period which suggests that this human retreat to Africa could be forced by climatic conditions. The low sea level in the Gibraltar Strait at that time could also facilitate the Iberian Peninsula colonization. The northwestern African M1c and the probable north central M1b expansions are coincidental with the Iberomaurusian and Capsian industries. The anomalous evolution of M1a2 lineages left the coalescence ages of the eastern Africa M1a expansion uncertain, but as suggested for the sister U6a1 radiation; these movements could be correlated in time with an African origin and expansion of Afroasiatic languages [40]. Finally, from a maternal genetic perspective it seems that Neolithic occupation of the Sahara had both eastern and western influences.  Most probably other mtDNA lineages participated in this human back flow to Africa. It has been suggested that the North African X1 branch of the Euroasiatic haplogroup X could be one of them [63]. 

Whilst this paper was under review, a new paper also dealing with U6 and M1 haplogroups was published [53]. Haplogroup topologies and phylogeographic conclusions proposed by Olivieri et al. [53] are highly coincidental with those proposed by us in our previous paper on U6 [40] and in the present paper, dealing with M1. Regrettably, there are differences in nomenclature for M1. Whereas our M1 phylogeny adhered to that proposed previously by other authors [21], Olivieri et al. [53] chose to apply their own. Nevertheless, the diagnostic positions for the different M1 subhaplogroups allowed us to establish subhaplogroup homologies between the two works. Clearly their M1b subgroup (defined by transition 13111) corresponds to our M1c subgroup; their M1a2 subgroup (defined by transition 15884) corresponds to our M1b subgroup. Finally, their M1a1 subgroup (defined by transitions at 3705, 12346 and 16359) corresponds to our M1a subgroup. In addition to the reinforcing overlap of ideas, it is worthwhile mentioning the high coincidence for the coalescence ages of M1 and the majority of its subhaplogroups, when the same substitution rate [8] is used. Olivieri et al. [53] calculated a coalescence time estimate of 36.8 ± 7.1 ky for the entire haplogroup M1 that matches our estimate of 35.2 ± 7.1 ky. Our coalescence time for M1c (25.7 ± 6.6 ky) also overlaps with Olivieri et al. [53] haplogroup M1b (23.4 ± 5.6 ky). Likewise, the coalescence age calculated for our M1a subhaplogroup (22.6 ± 8.1 ky) is in the range of the Olivieri et al. [53] estimation for their M1a1 subhaplogroup (20.6 ± 3.4 ky). The only discrepancy is about the coalescence time estimate between our M1b subhaplogroup (13.7 ± 4.8 ky) that is younger than that calculated by Olivieri et al. [53] for their homologous M1a2 (24.0 ± 5.7 ky). As our calculations are based only on three lineages and that of Oliveri et al [53] on six, we think that their coalescence time estimation should be more accurate that ours. In fact, when time estimation is based on the eight different lineages (AFR-KI43 is common to both sets) a coalescence age of 20.6 ± 5.0 ky is obtained. Although with overlapping errors, these results, together with the relative ancestral positions of each subgroup in the phylogenetic tree (Fig. 1), would suggest that the northwestern M1c clade radiation was older than those for the ubiquitous M1b and the eastern M1a clades, as also proposed by Olivieri et al. [53].

## Conclusion

This study provides evidence that M1, or its ancestor, had an Asiatic origin. The earliest M1 expansion into Africa occurred in northwestern instead of northeastern areas; this early spread reached the Iberian Peninsula even affecting the Basques. The majority of the M1a lineages found outside and inside Africa had a more recent eastern Africa origin. Both western and eastern M1 lineages participated in the Neolithic colonization of the Sahara. The striking parallelism between subclade ages and geographic distribution of M1 and its North African U6 counterpart strongly reinforces this scenario. Finally, a relevant fraction of M1a lineages present today in the European Continent and nearby islands possibly had a Jewish instead of the commonly proposed Arab/Berber maternal ascendance.

## Methods

### Lineages

We have analyzed thirteen complete or nearly complete mtDNA sequences belonging to the M1 subhaplogroup. Eight of them are new sequences. Two were published previously [7] but have been partially re-sequenced to confirm some dubious diagnostic positions. One additional Asiatic [54] and two Ethiopian M1 sequences [55] are from other authors. In addition we analyzed 261 partial M1 sequences gathered, in a global search, from more than 38,713 published or unpublished subjects sequenced mainly for the mtDNA HVSI segment.

### Complete mtDNA sequencing

Complete mtDNA were amplified in 32 overlapping fragments with primers and PCR conditions previously described [7]. The same primers were utilized to directly sequence both strands of the fragments on an ABI 3100 analyzer using Big-Dye Terminator chemistry (Applied Biosystems). Sequence data were assembled and compared with the SeqScape software (Applied Biosystems), and all chromatograms were visually inspected.

### M1 phylogenetic analyses

Phylogenetic relationships among complete mtDNA sequences were established using the reduced median network algorithm [56]. In addition to the thirteen complete or nearly complete M1 sequences, a complete M Indian sequence [25], assigned to the Indian subhaplogroup M30, was used as an outgroup.

### M1 phylogeographic analyses

Relationships among the different M1 haplotypes were inferred using the reduced median network algorithm [56]. To resolve reticulations, the highly recurrent mutations, 16182C, 16183C, 16093 and the in M1 recurrent 16249, were half weighted.

### Differences in accumulated mutations among M1 branches

The non-parametric test, resampling probability estimates for the difference between the means of two independent samples ()  was used to calculate the significance level of accumulated mutations between the different M1 subclades. Synonymous vs. nonsynonymous substitutions between lineages were compared by the exact-Fisher test. Differences between mean number of substitutions for coding and non-coding regions were t-Student tested.

### M1 diversity and differentiation within and between areas

Arlequin package [57] was used to evaluate the M1 diversity within areas using molecular nucleotide diversity (π) and gene diversity (h). AMOVA was used to test population structure. Pairwise differences between areas were obtained by means of linearized F_ST_. Contingence tests were used to compare differences in M1 and M1a frequencies between groups.

### Time estimates

For the complete sequences only substitutions in the coding region, excluding indels, were taken into account. The mean number of substitutions per site to the most common ancestor (ρ) of each clade [58] was estimated, and converted into time using two substitution rates: 1.7 × 10^-8 ^[6] and 1.26 × 10^-8 ^[8]. For HVSI, the age of clusters or expansions was calculated as the mean divergence (ρ) from inferred ancestral sequence types [58] and converted into time by assuming that one transition within np 16090–16365 corresponds to 20,180 years [59]. The standard deviation of the ρ estimator was calculated as previously described [60]. Ages for HVSI of complete sequences were also independently calculated.

### Accesion numbers

The eight new complete mitochondrial DNA sequences are registered under GenBank accession numbers: DQ779925–32.

## Authors' contributions

All the authors carried out extensive sequencing and RFLP analysis, and actively participated in the analysis and discussion of the data. In addition, JML and AMG recompiled African and Eurasian M1 sequences from published data. All the authors read and approved the final manuscript.

## Supplementary Material

Additional file 1 Appendix 1. Geographic distribution of subhaplogroup M1. Click here for file

Additional file 2 Appendix 2. Reference list for the appendix 1 citations. Click here for file
