# Supplementary material for: Mitochondrial lineage M1 traces an early human backflow to Africa
Source: BMC Genomics. 2007 Jul 9;8:223. doi: 10.1186/1471-2164-8-223 (PMC1945034; doi:10.1186/1471-2164-8-223)
Supplement: Additional file 2 — Appendix 2. Reference list for the appendix 1 citations. [file 1471-2164-8-223-S2.doc]

1.- Al-Zahery N, Semino O, Benuzzi G, Magri C, Passarino G, Torroni A, Santachiara-Benerecetti AS. 2003. Y-chromosome and mtDNA polymorphisms in Iraq, a crossroad of the early human dispersal and of post-Neolithic migrations. Mol Phylogenet Evol 28(3):458-472

2.- Alzualde A, Izagirre N, Alonso S, Alonso A, Albarrán C, Azcarate A, de la Rúa C. 2006. Insights into the “isolation” of the Basques: mtDNA lineages from the historical site of Aldaieta (6th-7th centuries AD). Am J Phys Anthropol 130:394-404

3.- Baasner A, Schafer C, Junge A, Madea B. 1998.Polymorphic sites in human mtDNA control region sequences: population data and maternal inheritance. Foren Sci Int 98:169-178

4.- Baasner A, Madea B. 2000. Sequence polymorphisms of the mitochondrial DNA control region in 100 German caucasians. Forensic Sci 45(6):1343-1348

5.- Babalini C, Martinez-Labarga C, Tolk HV, Kivisild T, Giampaolo R, Tarsi T, Contini I, Barac L, Janicijevic B, Martinovic Klaric I, Pericic M, Sujoldzic A, Villems R, Biondi G, Rudan P, Rickards O. 2005. The population history of the Croatian linguistic minority of Molise (southern Italy): a maternal view. Eur J Hum Genet 13(8):902-912

6.- Baranov PO, Babenko VN, Ivanova AV, Kobzev VF, Romashchenko AG, Voevoda MI. Characteristics of the mitochondrial genome of Russian Germans.Genetika 1999. 35(2):249-254

7.- Behar DM, Hammer MF, Garrigan D, Villems R, Bonne-Tamir B, Richards M, Gurwitz D, Rosengarten D, Kaplan M, Pergola SD, Quintana-Murci L, Skorecki K. **2004. MtDNA evidence for a genetic bottleneck in the early history of the Ashkenazi Jewish population**. Eur J Hum Genet 12(5):355-365

8.- Belledi M, Poloni ES, Casalotti R, Conterio F, Mikerezi I, Tagliavini J, Excoffier L. 2000. Maternal and paternal lineages in Albania and the genetic structure of Indo-European populations Eur J Hum Genet 8(7):480-486

9.- Belyaeva O, Bermisheva M, Khrunin A, Slominsky P, Bebyakova N, Khusnutdinova E, Mikulich A, Limborska S. 2003. Mitochondrial DNA variations in Russian and Belorussian populations. Hum Biol 75(5):647-660

10.- Bermisheva M, Tambets R, Villems R, Khusnutdinova E. 2002. Diversity of mitochondrial DNA haplogroups in ethnic populations of the Volga-Ural regions. Mol Biol (Mosk) 36(6):802-812

11.- Bermisheva MA, Kutuev IA, Spitsyn VA, Villems R, Batyrova AZ, Korshunova TIu, Khusnutdinova EK. 2004. Analysis of mitochondrial DNA variation in the population of Oroks. Hum Biol 76(6):877-900

12.- Bermisheva MA, Kutuev IA, Korshunova TIu, Dubova NA, Villems R, Khusnutdinova EK. 2004. Phylogeografic analysis of mitochondrial DNA Nogays: the high level of mixture of maternal lineages from Eastern and Western. Mol Biol (Omsk)38(4):617-624

13.- Bertranpetit J, Sala J, Calafell F, Underhill PA, Moral P, Comas D. 1995. Human mitochondrial DNA variation and the origin of the Basques. Ann Hum Genet 59:63-81

14.- Bini C, Ceccardi S, Luiselli D, Ferri G, Pelotti S, Colalongo C, Falconi M, Pappalardo G. 2003. Informativeness of the three hypervariable mitochondrial DNA regions in the population of Bologna (Italy). Forensic Sci Int 135(1):48-52

15.- Bosch E, Calafell, González-Neira A, Flaiz C, Mateu E, Scheil HG, Huckenbeck W, Efremovska L, Mikerezi I, Xirotiris N, Grasa C, Schmidt H, Comas D. (on line). Paternal and maternal lineages in the Balkans show a homogeneous landscape over linguistic barriers, except for the isolated Aromuns. Ann Hum Genet

16.- Brakez Z, Bosch E, Izaabel H, Akhayat O, Comas D, Bertranpetit J, Calafell F. 2001. Human mitochondrial DNA sequence variation in the Moroccan population of the Souss area. Ann Hum Biol 28(3):295-307

17.- Brandstätter A, Peterson CT, Irwin JA, Mpoke S, Koech DK, Parson W, Parsons TJ. 2004. Mitochondrial DNA control region sequences from Nairobi (Kenya): inferring phylogenetic parameters fro the establishment of a forensic database. Int J legal Med 118:294-306

18.- Brehm A, Pereira L, Bandelt H-J, Prata MJ, Amorim A. 2002. Mitochondrial portraits of the Cabo Verde: the Senegambian outpost of Atlantic slave trade. Ann Hum Genet 66:49-60

19.- Brehm A, Pereira L, Kivisild T, Amorim A. 2003. Mitochondrial portraits of the Madeira and Açores archipelagos witness different genetic pools of its settlers. Hum Genet 114:77-86

20.- Calafell F, Underhill P, Tolun A, Angelicheva D, Kalaydjeva L. 1996. From Asia to Europe: mitochondrial DNA sequence variability in Bulgarians and Turks. Ann Hum Genet 60:35-49

21.- Cali F, Le Roux MG, D'Anna R, Flugy A, De Leo G, Chiavetta V, Ayala GF, Romano V.J. 2001. MtDNA control region and RFLP data for Sicily and France. Legal Med114(4-5):229-231

22.- Casas MJ, Hagelberg E, Fregel R, Larruga JM, González AM.(on line). Human mitochondrial DNA diversity in an archaeological site in *al-Andalus*. Genetic impact of migrations from North Africa in medieval Spain. Am J Phys Anthropol

23.- CEPH database

24.- Cerny V, Hajek M, Cmejla R, Bruzek J, Brdicka R. 2004. mtDNA sequences of Chadic-speaking populations from northern Cameroon suggest their affinities with eastern Africa. Ann Hum Biol 31(5):554-569

25.- Chen Y-S, Olckers A, Schurr TG, Kogelnik AM, Huoponen K, Wallace DC. 2000. mtDNA variation in the South African Kung and Khwe and their genetic relationships to other african populations. Am J Hum Genet 66:1362-1383

26.- Cherni L, Loueslati BY, Pereira L, Ennafaa H, Amorim A, El Gaaied AB. 2005. Female gene pools of Berber and Arab neighboring communities in central Tunisia: microstructure of mtDNA variation in North Africa. Hum Biol 77(1):61-70

27.- Coia V, Destro-Bisol G, Verginelli F, Battaggia C, Boschi I, Cruciani F, Spedini G, Comas D, Calafell F. 2005. mtDNA variation in North Cameroon: lack of Asian lineages and implications for back migration from Asia to sub-Saharan Africa. Am J Phys Anthropol 128(3):678-681

28.- Comas D, Calafell F, Mateu E, Perez-Lezaun A, Bertranpetit J. 1996.Geographic variation in human mitochondrial DNA control region sequence: the population history of Turkey and its relationship to the European populations. Molec Biol Evol 13:1067-1077

29.- Comas D, Calafell F, Mateu E, Perez-Lezaun A, Bosch E, Martinez-Arias R, Clarimon J, Facchini F, Fiori G, Luiselli D, Pettener D, Bertranpetit J. 1998. Trading genes along the silk road: mtDNA sequences and the origin of central Asian populations. Am J Hum Genet 63(6):1824-1838

30.- Comas D, Calafell F, Bendukidze N, Fananas L, Bertranpetit J. 2000.Georgian and kurd mtDNA sequence analysis shows a lack of correlation between languages and female genetic lineages. Am J Phys Anthropol 112(1):5-16

31.- Corte-Real HB, Macaulay VA, Richards MB, Hariti G, Issad MS, Cambon-Thomsen A, Papiha S, Bertranpetit J, Sykes BC. 1996. Genetic diversity in the Iberian Peninsula determined from mitochondrial sequence analysis. Ann Hum Genet 60:331-350

32.- Crespillo M, Luque JA, Paredes M, Fernández R, Ramírez E, Valverde JL. 2000. Mitochondrial DNA sequences for 118 individuals from northeastern Spain. Int J Legal Med 114:130-132

33.- Derbeneva OA, Starikovskaya EB, Wallace DC, Sukernik RI. 2002. Traces of early Eurasians in the Mansi of northwest Siberia revealed by mitochondrial DNA analysis. Am J Hum Genet 70(4):1009-1014

34.- Derbeneva OA, Starikovskaya EB, Volodko NV, Wallace DC, Sukernik RI. 2002. Mitochondrial DNA variation in the Kets and Nganasans and its implications for the initial peopling of northern Eurasia. Russ Hum Genet 38(11).1554-1560

35.- Derenko MV, Grzybowski T, Malyarchuk BA, Dmbueva IK, Denisova GA, Czarny J, Dorzhu CM, Kakpakov VT, Míscicka- Sliwka D, Wozniak M, Zakharov IA. 2003. Diversity of mitochondrial DNA lineages in South Siberia. Ann Hum Genet 67:391-411

36.- Destro-Bisol G, Coia V, Boschi I, Verginelli F, Caglià A, Pascali V, Spedini G, Calafell F. 2004.The Analysis of variation of mtDNA Hypervariable region 1 suggest that eastern and western Pygmies diverged before the Bantu Expansion. The American Naturalist 163 (2):212-226

37.- Di Benedetto G, Erguven A, Stenico M, Castri L, Bertorelle G, Togan I, Barbujani G. 2001. DNA diversity and population admixture in Anatolia. Am J Phys Anthropol 115(2):144-156

38.- Dimo-Simonin N, Grange F, Taroni F, Brandt-Casadevall C, Mangin P. 2000. Forensic evaluation of mtDNA in a population from south west Switzerland. Int J Legal Med 113:89-97

39.- Di Rienzo A, Wilson AC. 1991.Branching pattern in the evolutionary tree for human mitochondrial DNA. Proc Nat Acad Sci USA 88:1597-1601

40.- Dubut V, Chollet L, Murail P, Cartault F, Beraud-Colomb E, Serre M, Mogentale-Profizi.2004. DNA polymorphisms in five French groups: importance of regional sampling.Eur J Hum Genet 12(4):293-300

41.- Dupuy BM, Olaisen B. 1996.mtDNA sequences in the Norwegian Saami and main populations. Advances in Forensic Haemogenetics 6:23-25

42.- Fadhlaoui-Zid K, Plaza S, Calafell F, Ben Amor M, Comas D, Bennamar El gaaied A. 2004. Mitochondrial DNA heterogeneity in Tunisian Berbers. Ann Hum Genet 68 (3):222-233

43.- Falchi A, Giovannoni L, Calo CM, Piras IS, Moral P, Paoli G, Vona G, Varesi L. 2006. Genetic history of some western Mediterranean human isolates through mtDNA HVR1 polymorphisms. J Hum Genet 51(1):9-14

44.- Fedorova SA, Bermisheva MA, Villems R, Maksimova NR, Khusnutdinova EK. 2003.

Analysis of mitochondrial DNA haplotypes in yakut population. Mol Biol (Mosk) 37(4):643-53

45.- Forster P, Cali F, Rohl A, Metspalu E, D'Anna R, Mirisola M, De Leo G, Flugy A, Salerno A, Ayala G, Kouvatsi A, Villems R, Romano V. 2002. Continental and subcontinental distributions of mtDNA control region types Int J Legal Med 116(2):99-108

46.- Francalacci P, Bertranpetit J, Calafell F, Underhill P. 1996. Sequence Diversity of the Control Region of Mitochondrial DNA in Tuscany and Its Implications for the Peopling of Europe. Am J Phys Anthropol 100:443-460

47.- Fraumene, C., Petretto, E., Pirastu, A.A.M. Striking differentiation of sub-populations within a genetically homogeneous isolate (Ogliastra) in Sardinia as revealed by mtDNA analysis. Hum Genet 2003.114:1-10

48.- González AM, Brehm A, Perez JA, Maca-Meyer N, Flores C, Cabrera VM. 2003. Mitochondrial DNA affinities at the Atlantic fringe of Europe. Am J Phys Anthropol 120(4):391-4043

49.- González AM, Cabrera, VM, Larruga JM, Tounkara A, Noumsi G, Thomas B, Moulds JM. (on line). Mitochondrial DNA variation in Mauritania and Mali and their genetic relationship to other western Africa populations. Ann Hum Genet

50.- Graven L, Passarino G, Semino O, Boursot P, Santachiara-Benerecetti S, Langaney A, Excoffier L. 1995. Evolutionary correlation between control region sequence and restriction polymorphisms in the mitochondrial genome of a large Senegalese Mandenka sample.

Mol Biol Evol 12(2):334-345

51.- Gresham D, Morar B, Underhill PA, Passarino G, Lin AA, Wise C, Angelicheva D, Calafell F, Oefner PJ, Shen P, Tournev I, de Pablo R, Kucinskas V, Perez-Lezaun A, Marushiakova E, Popov V, Kalaydjieva L. 2001.Origins and divergence of the Roma (gypsies). Am J Hum Genet 69(6):1314-1331

52.- Helgason A, Sigurethardottir S, Gulcher JR, Ward R, Stefansson K. 2000. mtDNA and the origin of the Icelanders: deciphering signals of recent population history. Am J Hum Genet 66(3):999-1016

53.- Helgason A, Hickey E, Goodacre S, Bosnes V, Stefansson K, Ward R, Sykes B. 2001. mtDna and the islands of the North Atlantic: estimating the proportions of Norse and Gaelic ancestry. Am J Hum Genet 68(3):723-737

54.- Hofmann S, Jaksch M, Bezold R, Mertens S, Aholt S, Paprotta A, Gerbitz KD. 1997. Population genetics and disease susceptibility: characterization of central European haplogroups by mtDNA gene mutations, correlation with D loop variants and association with disease. Hum Mol Genet 6(11):1835-1846

55.- Jackson BA, Wilson JL, Kirbah S, Sidney SS, Rosenberg J, Bassie L, Alie JAD, McLean DC, Garvey TW, Ely B. 2005. Mitochondrial DNA genetic diversity among four ethnic groups in Sierra Leone. Am J Phys Anthropol 128(1):156-163

56.- Kalaydjieva L, Calafell F, Jobling MA, Angelicheva D, de Knijff P, Rosser ZH, Hurles ME, Underhill P, Tournev I, Marushiakova E, Popov V. 2001. Patterns of inter- and intragroup genetic diversity in the Vlax Roma as revealed by Y chromosome and mitochondrial DNA lineages. Eur J Hum Genet 9:97-104

57.- Kittles RA, Bergen AW, Urbanek M, Virkkunen M, Linnoila M, Goldman D, Long JC. 1999.Autosomal, mitochondrial, and Y chromosome DNA variation in Finland: evidence for a male-specific bottleneck. Am J Phys Anthropol 108(4):381-399

58.- Kivisild T, Bamshad MJ, Kaldma K, Metspalu M, Metspalu E, Reidla M, Laos S, Parik J, Watkings WS, Dixon ME., Papiha SS, Mastana SS, Mir MR, Ferac V, Villems R. 1999. Deep common ancestry of Indian and western-Eurasian mitochondrial DNA lineages. Curr Biol 9:1331-1334

59.- Kivisild T, Rootsi S, Metspalu M, Mastana S, Kaldma K, Parik J, Metspalu E, Adojaan M, Tolk H-V, Stepanov V, Gölge M, Usanga E, Papiha SS, Cinnioğlu C, King R, Cavalli-Sforza L, Underhill PA, Villems R. 2003. The Genetic Heritage of the Earliest Settlers Persists Both in Indian Tribal and Caste Populations. Am J Hum Genet 72(2): 313–332

60.- Kivisild T, Reidla M, Metspalu E, Rosa A, Brehm A, Pennarun E, Parik J, Geberhiwot T, Usanga E, Villems R. 2004. Ethiopian Mitochondrial DNA Heritage: Tracking Gene Flow Across and Around the Gate of Tears. Am J Hum Genet 75:752-770

61.- Kong Q-P, Yao Y-G, Liu M, Shen S-P, Chen C, Zhu C-L, Palanichamy MG, Zhang Y-P. 2003. Mitochondrial DNA sequence polymorphisms of five ethnic populations from northern China. Hum Genet 113:391-405

62.- Kouvatsi A, Karaiskou N, Apostolidis A, Kirmizidis G. 2001. Mitochondrial DNA sequence variation in Greeks. Hum Biol 73(6):855-869

63.- Krings M, Halim Salem A, Bauer K, Geisert H, Malek AK, Chaix L, Simon C, Welsby D, Di Rienzo A, Utermann G, Sajantila A, Pääbo S, Stoneking M. 1999. mtDNA analysis of Nile valley populations: A genetic corridor or a barrier to migration? Am J Hum Genet 64(4):1166-1176

64.- Lahermo P, Sajantila A, Sistonen P, Lukka M, Aula P, Peltonen L, Savontaus ML. 1996. The genetic relationship between the Finns and the Finnish Saami (Lapps): analysis of nuclear DNA and mtDNA. Am J Hum Genet 58:1309-1322

65.- Larruga JM, Diez F, Pinto FM, Flores C, González AM. 2001. Mitochondrial DNA characterisation of European isolates: the Maragatos from Spain. Eur J Hum Genet 9(9):708-716

66.- Lutz S, Weisser H-J, Heizmann J, Pollak S. 1998. Location and frequency of polymorphic positions in the mtDNA control region of individuals from Germany.  Int J Legal Med 111:67-77

67.- Maca-Meyer N, Sánchez-Velasco P, Flores C, Larruga JM, González AM, Oterino A, Leyva-Cobian F. 2003. Y chromosome and mitochondrial DNA characterization of Pasiegos, a human isolate from Cantabria (Spain). Ann Hum Genet 67:329-339

68.- Maca-Meyer N, Arnay M, Rando JC, Flores C, González AM, Cabrera VM and Larruga JM. 2004. Ancient mtDNA analysis and the Origin of the Guanches. Eur J Hum Genet 12: 155-162

69.- Maca-Meyer N, Cabrera VM, Arnay M, Flores C, Fregel R, González AM, Larruga JM. 2005. Mitochondrial DNA diversity in the 17th-18th Century remains from Tenerife (Canary Islands). Am J Phys Anthropol 127:418-426

70.- Macaulay V, Richards M, Hickey E, Vega E, Cruciani F, Guida V, Scozzari R, Bonné-Tamir B, Sykes B, Torroni A. 1999. The emerging tree of West Eurasian mtDNAs: a synthesis of control-region sequences and RFLPs. Am J Hum Genet 64: 232-249

71.- Malyarchuk BA, Derenko MV. 2001. Mitochondrial DNA variability in Russians and Ukrainians: implication to the origin of the Eastern Slavs. Ann Hum Genet 65:63-78

72.- Malyarchuk BA, Grzybowski T, Derenko MV, Czarny J, Wozniak M, Miscicka-Sliwka D. 2002. Mitochondrial DNA variability in Poles and Russians. Ann Hum Genet 66:261-283

73.- Malyarchuk BA, Grzybowski T, Derenko MV, Czarny J, Drobnic K, Miscicka-Sliwka D. 2003. Mitochondrial DNA variability in Bosnians and Slovenians.Ann Hum Genet 67:412-425

74.- Malyarchuk BA, Derenko MV, Grzybowski T, Lunkina A, Czarny J, Rynchkov S, Morozova I, Denisova G, Miscicka-Sliwka D. 2004. Differentiation of mitochondrial DNA and Y chromosomes in Russian populations. Hum Biol 76(6):887-900

75.- Martinez-Jarreta B, Prades A, Calafell F, Budowle B. Mitochondrial DNA HVI and HVII variation in a north-east Spanish population. Forensic Sci 2000. 45(5):1162-3

76.- Mateu E, Comas D, Calafell F, Pérez-Lezaun A, Abade A, Bertranpetit J. 1997. A tale of two islands: population history and nitochondrial DNA sequence variation of Bioko and Sao Tome, Gulf of Guinea. Ann Hum Genet 61:507-518

77.- McEvoy B, Richards M, Forster P, Bradley DG. 2004. The Longue Duree of genetic ancestry: multiple genetic marker systems and Celtic origins on the Atlantic facade of Europe. Am J Hum Genet 75(4):693-702

78.- Meinila M, Finnila S, Majamaa K. 2001. Evidence for mtDNA admixture between the Finns and the Saami. Hum Hered 52(3):160-170

79.- Mergen H, Öner R, Öner C. 2004. Mitochondrial DNA sequence variation in the Anatolian Peninsula (Turkey). J Genet 83(1):101-109

80.- Metspalu M, Kivisild T, Metspalu E, Parik J, Hudjashov G, Kaldma K, Serk P, Carmín M, Behar DM, Gilbert MTP, Endicott P, Mastana S, Papiha SS, Skorecki K, Torroni A,Villems R. 2004. Most of the extant mtDNA boundaries in South and Southwest Asia were likely shaped during the initial settlement of Eurasia by anatomically modern humans BMC Genet 2004; 5: 26

81.- Miller KWP. 1996. Molecular Genetic Analysis of Human Populations in Orkney and the North Atlantic Region. University of Cambridge

82.- Mogentale-Profizi N, Chollet L, Stevanovitch A, Dubut V, Poggi C, Pradie MP, Spadoni JL, Gilles A, Beraud-Colomb E. 2001. Mitochondrial DNA sequence diversity in two groups of Italian Veneto speakers from Veneto. Ann Hum Genet 65:153-166

83.- Mountain JL, Hebert JM, Bhattacharyya S, Underhill PA, Ottolenghi C, Gadgil M, Cavalli-Sforza LL. 1995. Demographic history of India and mtDNA-sequence diversity. Am J Hum Genet 56(4):979-992

84.- Nasidze I, Stoneking M. 2001. Mitochondrial DNA variation and language replacements in the Caucasus. Proc R Soc Lond 268:1197-1206

85.- Nasidze I, Ling ES, Quinque D, Dupanloup I, Cordaux R, Rychkov S, Naumova O, Zhukova O, Sarraf-Zadegan N, Naderi GA, Asgary S, Sardas S, Farhud DD, Sarkisian T, Asadov C, Kerimov A, M. Stoneking M. 2004. Mitochondrial DNA and Y-chromosome variation in the Caucasus. Ann Hum Genet 68: 205-221

86.- Nasidze I, Quinque D, Dupanloup I, Rychkov S, Naumova O, Zhukova O, Stoneking M. 2004. Genetic evidence concerning the origins of South and North Ossetians. Ann Hum Genet 68: 588-599

87.- Nasidze I, Quinque D, Ozturk M, Benndukidze N, Stoneking M. 2005. MtDNA and Y-chromosome variation in the Kurdish groups. Ann Hum Genet 69:401-412

88.- Opdal SH, Rognum TO, Vege A, Stave AK, Dupuy BM, Egeland T. 1998. Increased number of substitutions in the D-loop of mitochondrial DNA in the sudden infant death syndrome. Acta Paediatr 87(10):1039-1044

89.- Orekhov V, Poltoraus A, Zhivotovsky LA, Spitsyn V, Ivanov P, Yankovsky N. 1999. Mitochondrial DNA sequence diversity in Russians.FEBS Lett 445(1):197-201

90.- Pakendorf B, Wiebe V, Tarskaia LA, Spitsyn VA, Soodyall H, Rodewald A, Stoneking M. 2003. Mitochondrial DNA evidence for admixed origins of central Siberian populations. Am J Phys Anthropl 120(3):211-224

91.- Parson W, Parsons TJ, Scheithauer R, Holland MM. 1998. Population data for 101 Austrian Caucasian mitochondrial DNA d-loop sequences: application of mtDNA sequence analysis to a forensic case. Int J Legal Med 111:124-132

92.- Passarino G, Cavalleri GL, Lin AA, Cavalli-Sforza LL, Borresen-Dale AL, Underhill PA. 2002. Different genetic components in the Norwegian population revealed by the analysis of mtDNA and Y chromosome polymorphisms. Eur J Hum Genet 10(9):521-9

93.- Pereira L, Macaulay V, Torroni A, Scozzari R, Prata M-J, Amorin A. 2001. Prehistoric and historic traces in the mtDNA of Mozambique: insighys into the Bantu expansions and the slave trade. Ann Hum Genet 65:439-458

94.- Pereira L, Cunha C, Amorim A. 2004. Predicting sampling saturation of mtDNA haplotypes: an application to an enlarged Portuguese database. Int J Legal Med 118(3):132-136

95.- Pfeiffer H, Brinkmann B, Huhne J, Rolf B, Morris AA, Steighner R, Holland MM, Forster P. 1999. Expanding the forensic German mitochondrial DNA control region database: genetic diversity as a function of sample size and microgeography. Int J Legal Med 112:291-298

96.- Pfeiffer H, Forster P, Ortmann C, Brinkmann B. 2001. The results of an mtDNA study of 1,200 inhabitants of a German village in comparison to other Caucasian databases and its relevance for forensic casework Int J Legal Med 114(3):169-172

97.- Picornell A, Gomez-Barbeito L, Tomas C, Castro JA, Ramon MM. 2005. Mitochondrial DNA HVRI variation in Balearic populations. Am J Phys Anthropol 128(1):119-130

98.- Piercy R, Sullivan K, Benson N, Gill P. 1993. The application of mitochondrial DNA typing to the study of white Caucasian genetic identification. Int J Leg Med 106:85-90

99.- Pinto F, González AM, Hernández M, Larruga JM, Cabrera VM. 1996. Genetic relationship between the Canary Islanders and their African and Spanish ancestros inferred from mitochondrial DNA sequences. Ann Hum Genet 60:321-330

100.- Plaza S, Calafell F, Helal A, Bouzerna N, Lefranc G, Bertranpetit J, Comas D. 2003. Joining the pillars of Hercules: mtDNA sequences show multidirectional gene flow in the western Mediterranean. Ann Hum Genet 67:312-328

101.- Plaza S, Salas A, Calafell F, Corte-Real F, Bertranpetit J, Carracedo A, Comas D. 2004. Insights into the western Bantu dispersal: mtDNA lineage analysis in Angola. Hum Genet 115:439-447

102.- Pliss L, Tambets K, Loogväli E-L, Pronina N, Lazdins M, Krumina A, Baumanis V, Villems R.(on line). Mitochondrial DNA portrait of Latvians: Towards the understanding of the genetic structure of Baltic-speaking populations. Ann Hum Genet

103.- Poetsch M, Wittig H, Krause D, Lignitz E. 2003.Mitochondrial diversity of a northeast German population sample. Forensic Sci Internat 137(2-3):125-132

104.- Pult I, Sajantila A, Simanainem J, Georgiev O, Schaffner W, Paabo S. 1994. Mitochondrial DNA sequences from Switzerland Reveal striking Homogeneity of European Populations. Biol Chen 375:837-840

105.- Quintana-Murci L, Semino O, Bandelt H-J, Passarino G, McElreavey K, Santachiara-Benerecetti AS. 1999. Genetic evidence of an early exit of Homo sapiens sapiens from Africa through eastern Africa. Nat Genet 23(4):437-441

106.- Quintana-Murci L, Chaix R, Wells RS, Behar DM, Sayar H, Scozzari R, Rengo C, Al-Zahery N, Semino O, Santachiara-Benerecetti AS, Coppa A, Ayub Q, Mohyuddin A, Tyler-Smith C, Qasim Mehdi S, Torroni A, McElreavey K. 2004. Where west meets east: the complex mtDNA landscape of the southwest and Central Asian corridor. Am J Hum Genet 74(5):827-845

107.- Rajkumar R, Kashyap VK. 2003. Mitochondrial DNA hypervariable region I and II sequence polymorphism in the Dravidian linguistic group of India. J Forensic Sci 48(1):227-237

108.- Rajkumar R, Kashyap VK. 2003. Haplotype diversity in mitochondrial DNA hypervariable regions I and II in three communities of Southern India. Forensic Sci Internat 136:79-82

109.- Rando JC, Pinto F, González AM, Hernández M, Larruga JM, Cabrera VM, Bandelt HJ. 1998. Mitochondrial DNA analysis of northwest African populations reveals genetic exchanges with European, near-eastern, and sub-Saharan populations. Ann Hum Genet 62:531-550

110.- Rando JC, Cabrera VM, Larruga JM, Hernández M, González AM, Pinto F, Bandelt HJ. 1999. Phylogeographic patterns of mtDNA reflecting the colonization of the Canary Islands.Ann Hum Genet 63:413-428

111.- Richards M, Corte-Real H, Forster P, Macaulay V, Wilkinson-Herbots H, Demaine A, Papiha S, Hedges R, Bandelt HJ, Sykes B. 1996. Paleolithic and Neolithic lineages in the European mitochondrial gene pool. Am J Hum Genet 59:185-203

112.- Richards M, Macaulay V, Hickey E, Vega E, Sykes B, Guida V, Rengo C, Sellitto D, Cruciani F, Kivisild T, Villems R, Thomas M, Rychkov S, Rychkov O, Rychkov Y, Golge M, Dimitrov D, Hill E, Bradley D, Romano V, Cali F, Vona G, Demaine A, Papiha S, Triantaphyllidis C, Stefanescu G, Hatina J, Belledi M, Di Rienzo A, Novelletto A, Oppenheim A, Norby S, Al-Zaheri N, Santachiara-Benerecetti S, Scozari R, Torroni A, Bandelt HJ. 2000. Tracing European founder lineages in the Near Eastern mtDNA pool.Am J Hum Genet 67(5):1251-1276

113.- Rosa A, Brehm A, Kivisild T., Metspalu, E., and Villems, R. 2004.MtDNA profile of west Africa Guineans: Towards a better understanding of the Senegambia region. Ann Hum Genet 68:340-352

114.- Rousselet F, Mangin P. 1998. Mitochondrial DNA polymorphisms: a study of 50 French Caucasian individuals and application to forensic casework. Int J Legal Med 111(6):292-298

115.- Sajantila A, Lahermo P, Anttinen T, Lukka M, Cistonen P, Savontaus M-L, Aula P Beckman L, Tranebjaerg L, Gedde-Dahl T, Issel-Tarver L, DiRienzo A, Paabo S. 1995. Genes and languages in Europe: an analysis of mitochondrial lineages. Genome Res 5:42-52

116.- Sajantila A, Salem AH, Savolainen P, Bauer K, Gierig C, Paabo S. 1996. Paternal and maternal DNA lineages reveal a bottleneck in the founding of the Finnish population. Proc Natl Acad Sci U S A 93(21):12035-12039

117.- Salas A, Comas D, Lareu MV, Bertranpetit J, Carracedo A. 1998. mtDNA analysis of the Galician population: a genetic edge of European variation. Eur J Hum Genet 6(4):365-375

118.- Salas A, Richards M, De la fe T, Lareu MV, Sobrino B, Sanchez-Diz P, Macaulay V, Carracedo A. 2002. The making of the African mtDNA landscape. Am J Hum Genet 71(5):1082-1111

119.- Sampietro ML, Caramelli D, Lao O, Calafell F, Comas D, Lari M, Agusti B, Bertranpetit J, Lalueza-Fox C. 2005. The genetics of the pre-Roman Iberian Peninsula: a mtDNA study of ancient Iberians. Ann Hum Genet 69:535-548

120.- Santos C, Lima M, Montiel R, Angles N, Pires L, Abade A, Aluja MP. 2003. Genetic Structure and Origin of Peopling in the Azores Islands (Portugal): The view from mtDNA. Ann Hum Genet 67:433-456

121.- Shen P, Lavi T, Kivisild T, Chou V, Sengun D, Gefel D, Shpirer I, Wolf E, Hillel J, Feldman MW, Oefner PJ. 2004. Reconstruction of patrilineages and matrilineages of Samaritans and other Israeli populations from Y-chromosome and mitochondrial DNA sequence variation. Hum Mutat 24(3):248-260

122.- Starikovskaya EB, Sukernik RI, Derbeneva OA, Volodko NV, Ruiz-Pesini E, Torroni A, Brown MD, Lott MT, Hosseini SH, Huoponen K, Wallace DC. 2005. Mitochondrial DNA diversity in indigenous populations of the southern extent of Siberia, and the origins of native American haplogroups. Ann Hum Genet 69:67-89

123.- Stenico M, Nigro L, Bertorelle G, Calafell F, Capitanio M, Corrain C, Barbujani G. 1996. High Mitochondrial DNA Sequence Diversity in Linguistic Isolates of the Alps. Am J Hum Genet 59:1363-1375

124.- Stevanovitch A, Gilles A, Bouzaid E, Kefi R, Paris F, Gayraud RP, Spadoni JL, El-Chenawi. 2003. Mitochondrial DNA Sequence diversity in a sedentary population from Egypt. Ann Hum Genet 68:23-39

125.- Tagliabracci A, Turchi C, Buscemi L, Sassaroli C. 2001. Polymorphism of the mitochondrial DNA control region in Italians Int J Legal Med114(4-5):224-228

126.- Tambets K, Kivisild T, Metspalu E, Parik J, Kaldma K, Laos S, Tolk H-V, Gölge M, Demirtas H, Geberhiwot T, Papiha SS, Franco de Stefano G, Villems R. 2000. The topology of the maternal lineages of the Anatolian and Trans-Caucasus populations and the peopling of Europe: some preliminary considerations In Archaeogenetics: DNA and the population prehistory of Europe. Edited by Colin Renfrew& Katie Boyle McDonald Institute for Archaeological Research. University of Cambridge. Cambridge

127.- Tanaka M, Cabrera VM, González AM, Larruga JM, Takeyasu T, Fuku N, Guo LJ, Hirose R, Fujita Y, Kurata M, Shinoda K-I,Umetsu K, Yamada Y, Oshida Y, Sato Y, Hattori N, Mizuno Y, Arai Y, Hirose N, Ohta S, Ogawa O, Tanaka Y, Kawamori R, Shamoto-Nagai M, Maruyama W, Shimokata H, Suzuki R, and Shimodaira H. 2004. Mitochondrial genome variation in Eastern Asia and the peopling of Japan. Genome Res 14:1832-1850

128.- Thomas MG, Weale ME, Jones AL, Richards M, Smith A, Redhead N, Torroni A, Scozzari R, Gratrix F, Tarekegn A, Wilson JF, Capelli C, Bradman N, Goldstein DB. 2002. Founding mothers of Jewish communities: geographically separated Jewish groups were independently founded by very few female ancestors. Am J Hum Genet 701411-1420

129.- Tolk HV, Pericic M, Barac L, Klaric IM, Janicijevic B, Parik J, Villems R, Rudan P. 2000. mtDNA haplogroups in the populations of Croatian Adriatic Islands. Coll Anthrop 24:267-279

130.- Tolk HV, Barac L, Pericic M, Klaric IM, Janicijevic B, Campbell H, Rudan I, Kivisild T, Villems R, Rudan P. 2001. The evidence of mtDNA haplogroup F in a European population and its ethnohistoric implications. Eur J Hum Genet 9(9):717-723

131.- Trovoada MJ, Pereira L, Gusmao L, Abade A, Amorim A, Prata MJ. 2003. Pattern of mtDNA variation in three populations from Sao Tomé e Príncipe. Ann Hum Genet 68:40-54

132.- Vanecek T, Vorel F, Sip M. 2004. Mitochondrial DNA D-loop hypervariable regions: Czech population data. Int. J. Legal Med 118:14-18

133.- Varesi L, Memmí M, Cristofari M-C, Mameli GE, Caló CM, Vona G. 2000. Mitochondrial control-region sequence variation in the Corsican population, France. Am J Hum Biol12:339-351

134.- Verginelli F, Donati F, Coia V, Boschi I, Palmirotta R, Battista P, Costantini RM, Destro-Bisol G. 2003. Variation of the hypervariable region-1 of mitochondrial DNA in central-eastern Italy. J Forensic Sci 48(2):443-444

135.- Vernesi C, Di Benedetto G, Caramelli D, Secchieri E, Simoni L, Katti E, Malaspina P, Novelletto A, Marin VTM, Barbujani G. 2001. Genetic characterization of the body attributed to the evangelist Luke. PNAS 98(23):13460-13463

136.- Vernesi C, Fuselli S, Castri L, Bertorelle G, Barbujani G. 2002. Mitochondrial diversity in linguistic isolates of the Alps: a reappraisal.Hum Biol 74(5):725-730

137.- Vigilant LA. 1990. Control region sequences from African populations and the evolution of human mitochondrial DNA. PhD thesis, University of California, Berkeley

138.- Vona G, Ghiani ME, Caló CM, Vacca L, Memmí M, Varesi L. 2001. Mitochondrial DNA variation sequence análisis in Sicily. Am J Hum Biol 13:576-589

139.- Wang W, Wise Ch, Baric T, Black T, Bittles AH. 2003. The origins and genetic structure of three co-resident Chinese Muslim populations: the Salar, Bo´an and Dongxians. Hum Genet 113:244-252

140.- Watson E, Bauer K, Aman R, Weiss G, von Haeseler A, Pääbo S. 1996. mtDNA sequence diversity in Africa. Am J Hum Genet 59:437-444

141.- Watson E, Foster P, Richards M, Bandelt H-J. 1997. Mitochondrial Footprints of Human Expansions in Africa. Am J Hum Genet 61:691-704

142.- Yacoubi-Loueslati B, Cherni L, Khodjet-el Khil H, Ennafaa H, Pereira L, Amorim A, Ben Ayed F, Ben Ammar Elgaaied A. 2006. Islands inside an island: reproductive isolates on Jerba Island. Am J Hum Biol 18:149-153

143.- Yao Y-G, Nie L, Harpending H, Fu Y-X, Yuan Z-G, and Zhang Y-P. 2002. Genetic relationship of Chinese ethnic populations revealed by mtDNA sequence diversity. Am J Phys Anthropol 118:63-76

144.- Zupanic Pajnic I, Balazic J, Komel R. 2004. Sequence polymorphism of the mtDNA control region in the Slovenian population. Int J Legal Med 118:1-4

145.- References in table 3 from Tanaka et al., 2004 (ref. 127)

146.- Unpublished data
